# Cap Polyposis Presenting with Large Bowel Obstruction Mimicking Advanced Colorectal Cancer: A Case Report

**DOI:** 10.70352/scrj.cr.26-0438

**Published:** 2026-08-13

**Authors:** Shodai Mizuno, Takashi Takenoya, Yusuke Asada, Mai Tsutsui, Ippei Oto, Koji Osumi, Kenichi Suzuki, Kaori Kameyama, Noriaki Kameyama

**Affiliations:** 1 Department of Surgery, Ogikubo Hospital, Tokyo, Japan; 2 Department of Surgery, Teikyo University School of Medicine, Tokyo, Japan; 3 Department of Gastroenterology, Ogikubo Hospital, Tokyo, Japan; 4 Department of Pathology, Showa University Northern Yokohama Hospital, Yokohama, Kanagawa, Japan

**Keywords:** cap polyposis, bowel obstruction, colorectal cancer, laparoscopic surgery, inflammatory colorectal disease

## Abstract

**INTRODUCTION:**

Cap polyposis is a rare, benign inflammatory colorectal disease that usually presents with diarrhea, mucous stool, or abdominal discomfort. Presentation with large bowel obstruction requiring surgical intervention is extremely uncommon.

**CASE PRESENTATION:**

A 42-year-old Japanese man presented with anal pain. Colonoscopy revealed a circumferential stenotic lesion in the sigmoid colon that prevented passage of the endoscope, and advanced colorectal cancer causing bowel obstruction was strongly suspected. A transanal ileus tube was inserted for decompression, followed by elective laparoscopic low anterior resection with a temporary diverting ileostomy. Histopathological examination unexpectedly demonstrated cap polyposis, characterized by elongated and distorted crypts, mucosal erosion, and fibrinopurulent exudates forming cap-like structures. The patient was also positive for *Helicobacter pylori* infection and underwent successful eradication therapy after surgery and remained recurrence-free during 18 months of follow-up.

**CONCLUSIONS:**

Cap polyposis presenting with bowel obstruction is extremely rare and may mimic advanced colorectal cancer. Surgical intervention can be clinically justified in cases where malignancy cannot be excluded preoperatively.

## Abbreviations

CA19-9
: carbohydrate antigen 19-9
CEA
: carcinoembryonic antigen
H. pylori
: Helicobacter pylori

## INTRODUCTION

Cap polyposis is a rare, benign inflammatory colorectal disease characterized by inflammatory polyps covered with fibrinopurulent exudates, forming cap-like structures.^1)^ The disease most commonly affects the rectum and sigmoid colon and typically presents with diarrhea, mucous stool, abdominal pain, or rectal bleeding.^1,2)^ Although the etiology remains unclear, abnormal colonic motility, chronic mucosal irritation, and *H. pylori* infection have been implicated in its pathogenesis.^3–5)^

Most cases of cap polyposis are managed conservatively, including bowel habit control, anti-inflammatory therapy, and *H. pylori* eradication therapy.^3–5)^ Surgical intervention is rarely required; however, severe obstructive lesions mimicking advanced colorectal cancer may necessitate resection for both diagnosis and treatment.^6,7)^

We herein report a rare case of cap polyposis presenting with large bowel obstruction and circumferential stenosis that was difficult to differentiate preoperatively from advanced colorectal cancer, resulting in laparoscopic surgical resection.

## CASE PRESENTATION

**Fig. 1:**
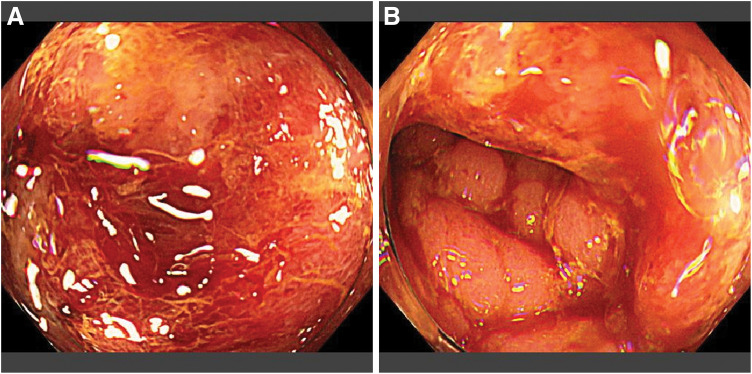
Sigmoid colon stenosis. (**A**) Circumferential stenosis with edematous and erythematous mucosa in the sigmoid colon. (**B**) Severe luminal narrowing preventing passage of the endoscope.

**Fig. 2:**
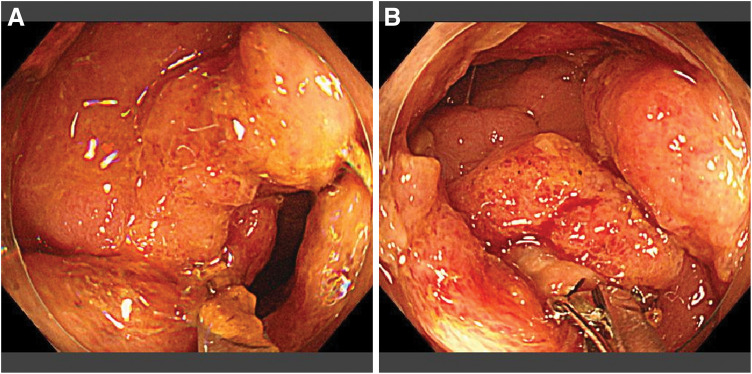
Type 2-like rectosigmoid lesions. (**A**) Type 2-like inflammatory lesion in the rectum. (**B**) Type 2-like inflammatory lesion in the rectosigmoid colon with edematous mucosa, mimicking advanced colorectal cancer.

A 42-year-old Japanese man presented with anal pain. He had no remarkable past medical history. Colonoscopy revealed a circumferential stenotic lesion with edematous and erythematous mucosa in the sigmoid colon that prevented passage of the endoscope (**Fig. 1**). Type 2-like inflammatory lesions with erythematous and edematous mucosa were observed from the rectum to the rectosigmoid colon (**Fig. 2**). Biopsy specimens demonstrated only inflammatory changes without evidence of malignancy.

**Fig. 3:**
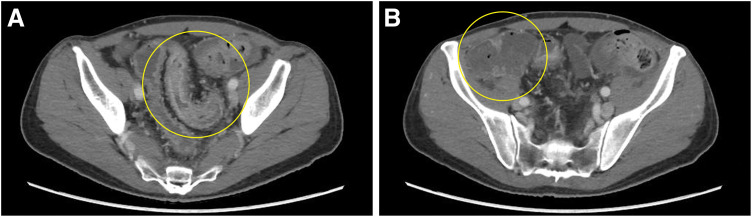
CT findings. (**A**) Circumferential wall thickening with enhancement extending approximately 10 cm in the sigmoid colon. (**B**) Marked dilation of the proximal colon was observed.

Contrast-enhanced CT revealed circumferential wall thickening extending approximately 10 cm in the sigmoid colon, accompanied by marked dilation of the proximal colon (**Fig. 3**). Advanced colorectal cancer causing large bowel obstruction was strongly suspected because of the severe circumferential stenosis and long-segment involvement. Serum CEA and CA19-9 levels were within the normal range (<1.73 ng/mL and 3.1 U/mL, respectively). A contrast study through the transanal ileus tube confirmed severe stenosis of the rectosigmoid colon.

A transanal ileus tube was inserted for decompression, followed by elective laparoscopic low anterior resection with a temporary diverting ileostomy.

**Fig. 4:**
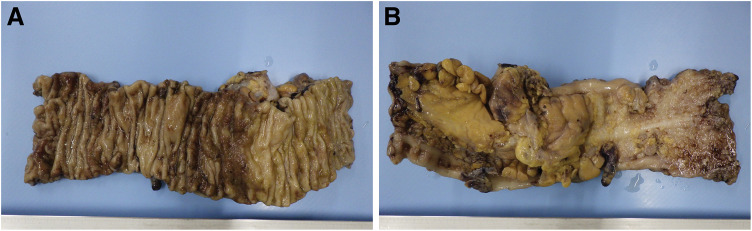
Macroscopic findings of the resected specimen. (**A**) Mucosal view of the resected rectosigmoid colon demonstrating circumferential inflammatory mucosal involvement with edema and mucosal irregularity. (**B**) Serosal view of the resected specimen. No discrete tumor mass was identified.

**Fig. 5:**
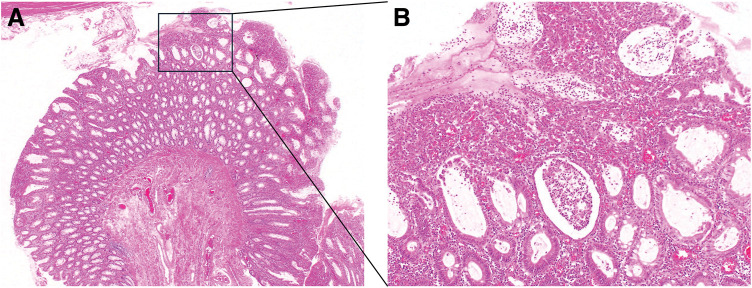
Histopathological findings. (**A**) Low-power view demonstrating inflammatory mucosal lesions with elongated and distorted crypts (hematoxylin and eosin staining). (**B**) High-power view showing mucosal erosion and fibrinopurulent exudates forming characteristic cap-like structures (hematoxylin and eosin staining).

**Fig. 6:**
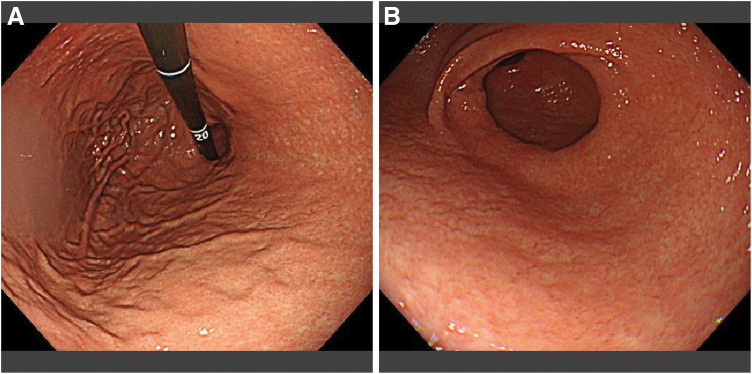
Upper gastrointestinal endoscopic findings. (**A**) Atrophic gastritis in the gastric body. (**B**) Atrophic mucosal changes in the antrum suggestive of *H. pylori* infection. *H. pylori, Helicobacter pylori*

Macroscopically, the resected specimen demonstrated circumferential inflammatory mucosal lesions with mucosal edema and irregularity (**Fig. 4**). No distinct tumor mass was identified. In addition, the degree of stenosis appeared less prominent than that suggested by the preoperative endoscopic and radiological findings. Histopathological examination revealed elongated and distorted crypts, mucosal erosion, and fibrinopurulent exudates forming characteristic cap-like structures (**Fig. 5**). No malignant findings were identified. Based on these findings, the patient was diagnosed with cap polyposis. Because previous reports have suggested an association between cap polyposis and *H. pylori* infection, upper gastrointestinal endoscopy was subsequently performed and demonstrated atrophic gastritis (**Fig. 6**). The patient tested positive for *H. pylori* infection. The postoperative course was uneventful. The patient subsequently underwent successful *H. pylori* eradication therapy and remained recurrence-free during 18 months of follow-up.

## DISCUSSION

Cap polyposis is a rare, nonneoplastic inflammatory colorectal disease first described in the 1980s.^1)^ The disease predominantly involves the rectum and sigmoid colon and typically presents with diarrhea, mucous stool, abdominal pain, and rectal bleeding.^1,2)^ Typical endoscopic findings include multiple sessile inflammatory polyps covered by fibrinopurulent exudates, usually localized in the rectum and sigmoid colon, while histopathological examination demonstrates elongated crypts and fibrinopurulent exudates forming characteristic cap-like structures.^1,2,8)^

In contrast, the present case demonstrated extensive circumferential stenosis, long-segment involvement, and type 2-like lesions associated with large bowel obstruction. Furthermore, the lesion lacked the typical appearance of multiple discrete inflammatory polyps. These atypical findings closely resembled advanced colorectal cancer and substantially contributed to the diagnostic difficulty.

Although the precise etiology remains unclear, several mechanisms have been proposed, including abnormal colonic motility, mucosal prolapse caused by excessive straining, chronic inflammation, and *H. pylori* infection.^3–5)^ Several reports have demonstrated regression of lesions after *H. pylori* eradication therapy, suggesting a possible association between the disease and bacterial infection.^3–5)^

Most patients with cap polyposis can be managed conservatively, and spontaneous resolution has also been reported.^6)^ Surgical treatment is generally reserved for refractory or recurrent disease. Presentation with severe bowel obstruction requiring surgery is extremely uncommon.^7)^

In the present case, preoperative differentiation from advanced colorectal cancer was particularly difficult because the lesion demonstrated severe circumferential stenosis, long-segment involvement, and obstructive findings on CT. Although endoscopic biopsy revealed only inflammatory changes, the possibility of malignancy could not be excluded because superficial biopsy sampling may fail to detect deeply invasive carcinoma in stenotic lesions. Therefore, surgical intervention was considered clinically justified.

Data regarding long-term outcomes after surgical treatment of cap polyposis remain limited because of the rarity of the disease. Previous surgical case reports have described symptom resolution without recurrence for approximately 1 year of follow-up. However, recurrence after treatment remains a clinical concern. In a recent retrospective study, recurrence was observed in 18 of 35 patients (51.4%) during a median follow-up period of 26 months.^9)^ Although the study included patients treated by both endoscopic and surgical approaches, these findings suggest that recurrence is not uncommon and that long-term surveillance is warranted. Therefore, postoperative follow-up, particularly by colonoscopy, appears important even after apparently curative treatment. In the present case, the patient remained recurrence-free during 18 months of follow-up after surgery.

An additional consideration in the present case is whether a definitive preoperative diagnosis could have been established using more extensive tissue acquisition techniques. In cases where conventional forceps biopsy is nondiagnostic, jumbo forceps biopsy may provide larger tissue samples and potentially improve diagnostic accuracy. Furthermore, endoscopic US-guided tissue acquisition from deeper layers could theoretically facilitate the exclusion of invasive carcinoma when malignancy remains a concern despite negative biopsy findings. If characteristic histopathological findings of cap polyposis, including elongated crypts and fibrinopurulent exudates, were successfully obtained, these techniques might contribute to a preoperative diagnosis.

However, in the present case, severe circumferential stenosis prevented passage of the endoscope and substantially limited further endoscopic evaluation. In addition, the patient had already developed clinically significant large bowel obstruction requiring decompression with a transanal ileus tube. Therefore, although additional tissue acquisition techniques might theoretically have improved diagnostic confidence, their feasibility and diagnostic yield were uncertain. Given the persistent concern for malignancy and the presence of obstructive symptoms, surgical resection was considered an appropriate management strategy.

If a definitive diagnosis of cap polyposis had been established preoperatively, conservative management, including *H. pylori* eradication therapy, might have been considered before surgical intervention. Previous reports have demonstrated the regression of cap polyposis following successful eradication therapy. Therefore, surgery may potentially be avoided in selected patients when a definitive diagnosis is established before the development of severe symptoms.

However, in the present case, the patient had already developed clinically significant large bowel obstruction requiring decompression with a transanal ileus tube. Furthermore, malignancy could not be completely excluded despite biopsy examinations. Under these circumstances, delaying definitive treatment in favor of empirical conservative therapy would have carried a risk of overlooking an underlying colorectal malignancy. Therefore, surgical resection was considered an appropriate management strategy.

Another important feature of this case was the presence of large bowel obstruction caused by circumferential inflammatory stenosis. Chronic inflammation and repeated mucosal injury may have contributed to progressive fibrosis and luminal narrowing, eventually resulting in obstructive symptoms.

To our knowledge, this is the first reported surgical case of cap polyposis presenting with large bowel obstruction mimicking advanced colorectal cancer. This case highlights the importance of considering benign inflammatory diseases in the differential diagnosis of obstructive colorectal lesions and demonstrates that surgical resection may be necessary when malignancy cannot be excluded preoperatively.

## CONCLUSIONS

Cap polyposis presenting with large bowel obstruction is extremely rare and may closely mimic advanced colorectal cancer. Surgical intervention can be clinically justified in patients with severe stenotic lesions when malignancy cannot be excluded preoperatively.

## DECLARATIONS

### Availability of data and materials

The datasets supporting the conclusions of this article are included within the article.
